# Etiological Spectrum, Clinical Characteristics, and Six-Month Treatment Outcomes of Refractory Rickets in Children: A Prospective Observational Cohort Study From Eastern India

**DOI:** 10.7759/cureus.114151

**Published:** 2026-08-07

**Authors:** Debajyoti Mukhopadhyay, Aditi A Jaiswal, Priya Dutta, Picaso Chowdhury

**Affiliations:** 1 Paediatrics, Apollo Gleneagles Hospitals, Kolkata, IND; 2 Community and Family Medicine, Ram Krishna Dharmarth Foundation (RKDF) Bhopal, Bhopal, IND; 3 Psychiatry, Institute of Neurosciences Kolkata, Kolkata, IND

**Keywords:** distal renal tubular acidosis, hypophosphatemic rickets, refractory rickets, treatment outcome, vitamin d dependent rickets

## Abstract

Background: Refractory rickets comprises a heterogeneous group of inherited and acquired disorders characterized by persistent clinical, biochemical, and radiological evidence of rickets despite adequate vitamin D and calcium therapy. Owing to limited data from Eastern India, this study aimed to characterize the etiological spectrum of refractory rickets, compare the clinical, biochemical, and radiological characteristics across different etiologies, and evaluate treatment outcomes following six months of etiology-specific therapy.

Methods: This hospital-based prospective observational cohort study included 32 consecutive children with refractory rickets attending a tertiary pediatric endocrine centre in Eastern India. Demographic, clinical, biochemical, radiological, and genetic data (where available) were collected. Children were classified into distal renal tubular acidosis (DRTA), proximal renal tubular acidosis (PRTA), hypophosphatemic rickets (HPR), and vitamin D-dependent rickets (VDDR). All participants received etiology-specific treatment and were followed for six months. Treatment outcomes included radiological healing, biochemical response, and height gain.

Results: DRTA was the commonest etiology (43.8%), followed by HPR (28.1%), VDDR (18.8%), and PRTA (9.4%). Short stature was universal, while failure to thrive (75.0%), delayed motor development (78.1%), and skeletal deformities (93.8%) were common across all groups. Metabolic acidosis was confined to DRTA and PRTA, hyperparathyroidism predominated in VDDR, and reduced tubular maximum reabsorption of phosphate per glomerular filtration rate (TmP/GFR) was significantly associated with HPR and PRTA (all *p*<0.001). Radiological healing was achieved in all children after six months; however, complete biochemical healing occurred in only 18.8%, while 46.9% had persistent biochemical abnormalities. Median height gain was greatest in DRTA (2.7 cm). Baseline serum alkaline phosphatase (ALP) correlated with six-month ALP (ρ=0.776, *p*<0.001), and baseline parathyroid hormone independently predicted biochemical response (β=1.276, R²=0.412, *p*<0.05).

Conclusions: DRTA was the predominant cause of refractory rickets in this cohort. Although clinical manifestations overlapped, characteristic biochemical abnormalities enabled accurate etiological differentiation. Etiology-specific therapy resulted in universal radiological healing but variable biochemical recovery, highlighting the importance of early diagnosis and individualized management.

## Introduction

Rickets is a metabolic bone disorder of childhood characterized by defective mineralization of the growing epiphyseal growth plates and osteoid matrix due to disturbances in calcium, phosphate, or vitamin D metabolism [1]. Although nutritional rickets has declined in developed countries, it remains a major public health problem in low- and middle-income countries because of vitamin D deficiency, inadequate calcium intake, limited sunlight exposure, and socioeconomic factors [2-4]. Untreated rickets may result in growth retardation, skeletal deformities, delayed motor development, fractures, and long-term disability [1,2].

Refractory rickets is defined as the persistence of clinical, biochemical, and radiological evidence of rickets despite adequate vitamin D and calcium therapy [1]. It comprises a heterogeneous group of inherited and acquired disorders affecting calcium or phosphate homeostasis, broadly classified into calcipenic and phosphopenic forms [2,5,6]. Owing to overlapping clinical manifestations, accurate diagnosis requires systematic biochemical and radiological evaluation, supplemented by genetic testing where available [5-8]. Because clinical manifestations frequently overlap among inherited and acquired disorders, early etiological diagnosis remains challenging in routine pediatric practice.

Previous studies from All India Institutes of Medical Sciences (AIIMS) and Western India have identified renal tubular acidosis, vitamin D-dependent rickets (VDDR), and hereditary hypophosphatemic rickets (HPR) as the predominant etiologies, although their relative frequencies vary across populations [7,9]. International studies have similarly reported marked etiological heterogeneity [5,10]. However, evidence from Eastern India remains limited. Therefore, this study aimed to characterize the etiological spectrum, clinical, biochemical, and radiological characteristics, and six-month treatment outcomes of refractory rickets in children attending a tertiary pediatric endocrine centre in Eastern India.

## Materials and methods

A hospital-based observational cross-sectional study was conducted over a period of 12 months (February 2022 to January 2023) in the Department of Paediatrics at a tertiary care referral centre for pediatric endocrinology in Eastern India. Children attending the Pediatric Endocrinology outpatient department with suspected refractory rickets were screened for eligibility. Refractory rickets was defined as the persistence of clinical features, biochemical abnormalities, and radiological evidence of rickets despite treatment with vitamin D (2,000 IU/day with 500 mg/day elemental calcium in infants aged less than one year, or 3,000-6,000 IU/day with 600-800 mg/day elemental calcium in children aged one or more years) for a minimum duration of three months, without radiological or biochemical evidence of healing [11]. Children who demonstrated biochemical and radiological healing following standard vitamin D therapy, those with chronic liver disease or malabsorption disorders (including celiac disease), and those receiving medications known to interfere with vitamin D metabolism were excluded from the study.

Sampling

The sample size was estimated using the single population proportion formula based on a 95% confidence level. In the absence of published data on the prevalence of refractory rickets in India, a prevalence of 4.5% reported for rickets in an Indian pediatric population by Banik et al. [12] was used for estimation. Using an absolute precision of 7%, the minimum required sample size was calculated to be 34 children. During the study period, all consecutive eligible children with refractory rickets attending the Pediatric Endocrinology Clinic were enrolled. Owing to the rarity of the condition, 32 children fulfilling the inclusion criteria were recruited and included in the final analysis.

Because refractory rickets is an uncommon disorder and no reliable prevalence estimates were available for sample size calculation, all consecutive eligible children presenting during the study period were enrolled. Therefore, the study represents a consecutive sample of eligible patients rather than a prevalence-based survey.

Data collection, study tools and parameters used

After obtaining informed consent, detailed demographic and clinical information was recorded using a predesigned structured proforma. A comprehensive clinical examination was performed, including anthropometric assessment (height and weight), evaluation of skeletal deformities, developmental milestones, family history, and associated clinical features such as polyuria. Laboratory investigations included serum calcium, phosphate, alkaline phosphatase (ALP), intact parathyroid hormone (PTH), 25-hydroxyvitamin D, renal function tests, serum electrolytes, blood gas analysis, and relevant urinary investigations. Standard radiographs of the wrists and knees were obtained at baseline and after six months of treatment to assess disease severity and monitor radiological response. Radiological healing was defined as the appearance of a dense provisional zone of calcification at the metaphysis with resolution of metaphyseal fraying and cupping on follow-up radiographs. Additional investigations, including genetic testing, were performed where clinically indicated. Genetic testing was available in eight of the 32 children (25.0%) and identified pathogenic variants involving CYP27B1, VDR, FGF23, and ATP6V1B1, which were used to support etiological classification where applicable.

Based on the clinical, biochemical, radiological, and genetic findings, patients were classified as having distal renal tubular acidosis (DRTA), proximal renal tubular acidosis (PRTA), HPR, VDDR, or other specific etiologies. Treatment was instituted according to the underlying diagnosis, and all patients were followed for six months. Biochemical healing was defined as normalization of serum ALP levels at follow-up, while partial biochemical healing was defined as a reduction of ≥50% in serum ALP from baseline without complete normalization. Treatment outcomes were evaluated in terms of biochemical response, radiological healing, and change in height over the six-month follow-up period.

Data analysis

Data were entered into Microsoft Excel (Redmond, WA, USA) and analyzed using IBM SPSS Statistics version 27.0 (IBM Corp., Armonk, NY, USA). Continuous variables were assessed for normality using the Shapiro-Wilk test and summarized as median with interquartile range (IQR) or mean ± standard deviation (SD), as appropriate. Categorical variables were expressed as frequencies and percentages.

Associations between categorical variables and the etiological diagnosis of refractory rickets were assessed using the Pearson's Chi-square test or Fisher's exact test, as appropriate. Spearman's rank correlation was used to evaluate the relationship between baseline serum ALP and ALP after six months of treatment, as well as between age at diagnosis and height gain following treatment. The relationship between baseline PTH levels and change in ALP (ΔALP) after six months was initially assessed using Pearson's correlation, followed by simple linear regression to determine the predictive value of baseline PTH on biochemical response. A two-tailed p-value <0.05 was considered statistically significant.

Ethical approval

The study was approved by the Institutional Ethics Committee (Approval No. TPA 12/PAED/2022), and written informed consent was obtained from the parents or legal guardians of all participants prior to enrolment.

## Results

Baseline characteristics of the study population

A total of 32 children with refractory rickets were included in the study. Most children were diagnosed between 37 and 72 months of age (43.8%), followed by 13-36 months (40.6%), while 15.6% were diagnosed after 72 months of age. No child was diagnosed during the first year of life. The study population comprised 18 (56.3%) females and 14 (43.7%) males. Parental consanguinity and a positive family history of rickets were present in 21.9% and 12.5% of children, respectively. At presentation, all children (100%) had short stature, 24 (75.0%) had failure to thrive, 25 (78.1%) exhibited delayed gross motor development, and 30 (93.8%) had skeletal deformities.

Etiological spectrum and clinico-biochemical characteristics

Among the 32 children with refractory rickets, DRTA was the most common etiology, accounting for 43.8% (14/32) of cases, followed by HPR (28.1%; 9/32), VDDR (18.8%; 6/32), and PRTA (9.4%; 3/32) (Table 1).

**Table 1:** Association of clinico-biochemical characteristics with the etiological diagnosis of refractory rickets *Statistically significant at p < 0.05 DRTA: distal renal tubular acidosis, PRTA: proximal renal tubular acidosis, HPR: hypophosphatemic rickets, VDDR: vitamin D-dependent rickets, TmP/GFR: tubular maximum reabsorption of phosphate per glomerular filtration rate

Variable	DRTA (n = 14)	HPR (n = 9)	PRTA (n = 3)	VDDR (n = 6)	χ²	p value
Failure to thrive, n (%)	11 (78.6)	5 (55.6)	3 (100.0)	5 (83.3)	3.15	0.372
Skeletal deformity, n (%)	12 (85.7)	9 (100.0)	3 (100.0)	6 (100.0)	2.72	0.433
Motor delay, n (%)	9 (64.3)	7 (77.8)	3 (100.0)	6 (100.0)	4.10	0.252
Family history of rickets, n (%)	2 (14.3)	2 (22.2)	0	0	1.19	0.551
Parental consanguinity, n (%)	2 (14.3)	1 (11.1)	1 (33.3)	3 (50.0)	4.10	0.252
Metabolic acidosis, n (%)	14 (100.0)	0	3 (100.0)	0	32.00	<0.001*
Hyperparathyroidism, n (%)	1 (7.1)	1 (11.1)	0	6 (100.0)	24.89	<0.001*
Low TmP/GFR, n (%)	2 (14.3)	9 (100.0)	3 (100.0)	2 (33.3)	22.11	<0.001*

Short stature was present in all children, while failure to thrive (75.0%), delayed motor development (78.1%), and skeletal deformities (93.8%) were common across all etiological groups. None of these clinical characteristics differed significantly between etiologies (Table 1).

Distinct biochemical patterns differentiated the underlying etiologies. Metabolic acidosis was confined to children with DRTA and PRTA, hyperparathyroidism predominated in VDDR, whereas low tubular maximum reabsorption of phosphate per glomerular filtration rate (TmP/GFR) was observed primarily in HPR and PRTA (all p<0.001) (Table 1).

All children with HPR and PRTA exhibited hypophosphatemia together with reduced TmP/GFR, indicative of renal phosphate wasting. Low TmP/GFR was also observed in a minority of children with DRTA (14.3%) and VDDR (33.3%), and its distribution differed significantly across etiological groups (p < 0.0001) (Table 1).

Genetic confirmation was available in eight (25.0%) children, including four with CYP27B1-associated VDDR1, one with a VDR-associated VDDR2A, two with FGF23-associated HPR, and one with ATP6V1B1-associated DRTA.

Radiological findings and treatment outcomes

Following six months of etiology-specific therapy, all children achieved radiological healing. Complete biochemical healing was observed in six (18.8%) children, whereas partial biochemical healing occurred in 11 (34.4%). Persistent biochemical abnormalities remained in 15 (46.9%) children (Table 2).

**Table 2:** Radiological findings and treatment outcomes after six months according to the etiological diagnosis of refractory rickets DRTA: distal renal tubular acidosis, PRTA: proximal renal tubular acidosis, HPR: hypophosphatemic rickets, VDDR: vitamin D-dependent rickets

Variable	DRTA (n=14)	HPR (n=9)	PRTA (n=3)	VDDR (n=6)	Total (N=32)
Radiological healing, n (%)	14 (100.0)	9 (100.0)	3 (100.0)	6 (100.0)	32 (100.0)
Complete biochemical healing*, n (%)	3 (23.1)	3 (33.3)	0	0	6 (18.8)
Partial biochemical healing†, n (%)	5 (38.5)	3 (33.3)	0	3 (50.0)	11 (34.4)
No biochemical healing, n (%)	6 (42.9)	3 (33.3)	3 (100.0)	3 (50.0)	15 (46.9)

**Figure 1:**
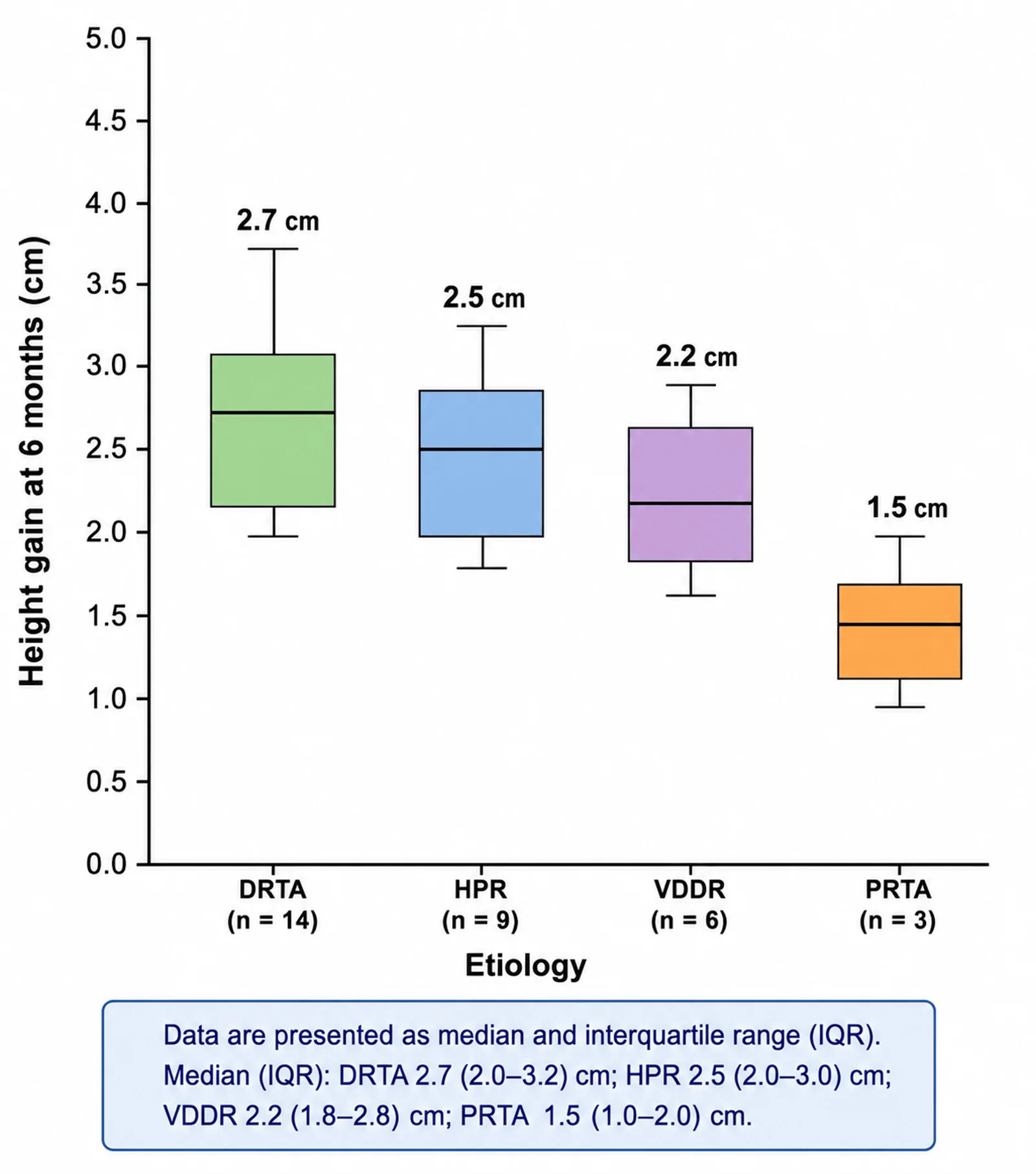
Box plot showing height gain after six months according to etiology DRTA: distal renal tubular acidosis, PRTA: proximal renal tubular acidosis, HPR: hypophosphatemic rickets, VDDR: vitamin D-dependent rickets

Median height gain ranged from 1.5 cm in PRTA to 2.7 cm in DRTA (Figure 1). Nephrocalcinosis was present in nine (28.1%) children, predominantly among those with DRTA.

Nephrocalcinosis was identified in nine of 32 children (28.1%) at diagnosis. Of these, seven (77.8%) had DRTA and two (22.2%) had HPR, indicating that nephrocalcinosis occurred predominantly among children with renal tubular disorders.

Baseline factors associated with six-month treatment outcomes

Baseline ALP correlated positively with ALP at six months (ρ=0.776, p<0.001). Age at diagnosis correlated with height gain (ρ=0.361, p=0.046). Baseline PTH correlated with change in ALP (r=0.642, p=0.021) and independently predicted biochemical response on linear regression (β=1.276, R²=0.412, p<0.05) (Table 3).

**Table 3:** Relationship between baseline clinical and biochemical parameters and six-month treatment outcomes *Statistically significant at p < 0.05

Baseline parameter	Six-month outcome	Analysis	Test statistics	p value
Serum alkaline phosphatase (ALP)	ALP at 6 months	Spearman's rank correlation	ρ = 0.776	<0.001*
Age at diagnosis	Height gain at 6 months	Spearman's rank correlation	ρ = 0.361	0.046*
Serum parathyroid hormone (PTH)	Change in ALP (ΔALP)	Pearson's correlation	r = 0.642	0.021*
Serum parathyroid hormone (PTH)	Change in ALP (ΔALP)	Linear regression	β = 1.276, R^2^= 0.412	<0.05*

## Discussion

DRTA was the predominant cause of refractory rickets (43.8%) in the present study, followed by HPR, VDDR, and PRTA. This distribution is consistent with previous Indian studies from tertiary referral centres, which have similarly identified renal tubular disorders as the leading causes of refractory rickets [10,13,14]. Comparable findings have been reported from Western India, although the relative frequency of hereditary hypophosphatemic disorders varies across centres, probably reflecting differences in referral patterns, genetic background, and healthcare access [15,16]. In contrast, international studies, particularly those from specialized genetic centres, have reported X-linked HPR as the predominant etiology [2,5]. This difference may partly reflect referral bias, as specialized genetic centres are more likely to receive children with suspected inherited metabolic bone disorders, thereby increasing the proportion of hereditary forms in their cohorts. In contrast, referral patterns, availability of genetic testing, and regional differences in disease spectrum may have influenced the etiological distribution observed in our study. Therefore, direct comparison between studies should be interpreted with caution.

Short stature, failure to thrive, delayed motor development, and skeletal deformities were the predominant clinical manifestations in our cohort, consistent with previous Indian studies [2,13,14]. Although failure to thrive and motor delay were more frequent among children with PRTA and VDDR, these differences were not statistically significant, reinforcing earlier observations that clinical features alone are insufficient to distinguish the underlying etiologies [15].

Distinct biochemical abnormalities facilitated etiological differentiation. Metabolic acidosis was confined to DRTA and PRTA, consistent with the recognized biochemical hallmark of renal tubular acidosis [16,17]. Hyperparathyroidism occurred exclusively in children with VDDR, reflecting impaired vitamin D metabolism and secondary hyperparathyroidism, as previously reported [2,6]. Similarly, reduced TmP/GFR in children with HPR and PRTA confirmed renal phosphate wasting and supports its utility as a reliable marker for differentiating phosphopenic from calcipenic rickets [5].

All children demonstrated radiological healing following six months of etiology-specific treatment, although complete biochemical healing was achieved in only 18.8%, indicating that biochemical recovery may lag behind radiological improvement, as reported previously [18]. Children with DRTA exhibited the greatest height gain, consistent with earlier studies demonstrating catch-up growth following correction of chronic metabolic acidosis with alkali therapy [17,19]. Nephrocalcinosis was identified predominantly among children with DRTA, in agreement with previous paediatric nephrology studies describing it as a common complication of chronic hypercalciuria [17].

Baseline serum ALP showed a strong positive correlation with ALP levels after six months, while baseline PTH independently predicted biochemical response, suggesting that the severity of biochemical derangement at presentation influences treatment outcomes. Similar observations have been reported in hereditary forms of rickets, although available evidence remains limited because of the rarity of these disorders [18,20]. Furthermore, the positive correlation between age at diagnosis and height gain underscores the importance of timely diagnosis and initiation of etiology-specific therapy to optimize long-term skeletal outcomes [5,13].

**Figure 2:**
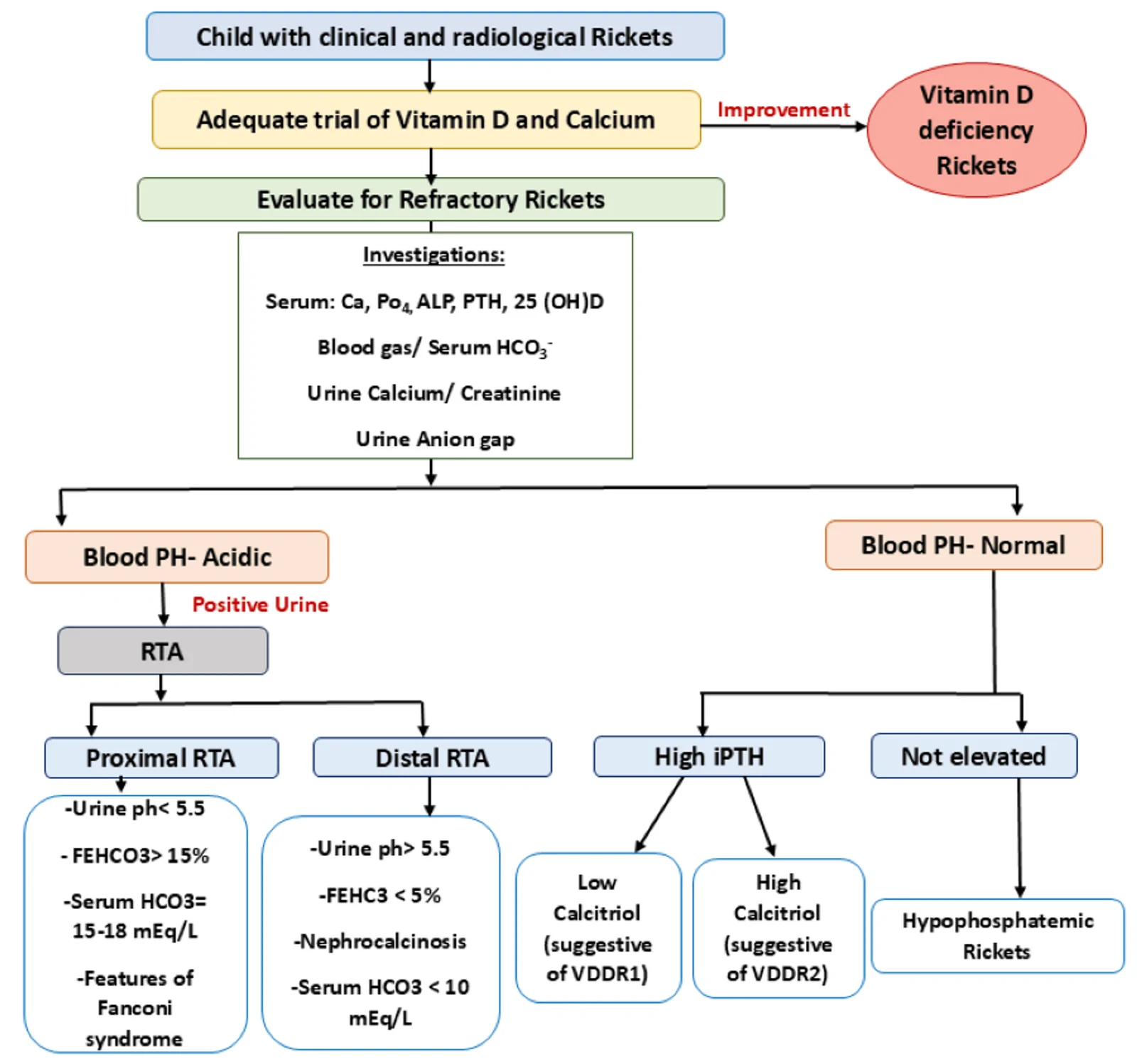
Proposed diagnostic algorithm for the evaluation of children with refractory rickets based on the clinico-biochemical findings observed in the present study. ALP: alkaline phosphatase, Ca: Calcium, FEHCO₃: fractional excretion of bicarbonate, HCO₃⁻: bicarbonate, iPTH: intact parathyroid hormone, PTH: parathyroid hormone, PO₄: phosphate, RTA: renal tubular acidosis, TmP/GFR: tubular maximum reabsorption of phosphate per glomerular filtration rate, VDDR1: vitamin D-dependent rickets type 1, VDDR2: vitamin D-dependent rickets type 2, 25(OH)D: 25-hydroxyvitamin D (calcidiol), mEq/L: milliequivalents per liter.

The present study demonstrates that DRTA is the predominant cause of refractory rickets in Eastern India. Despite overlapping clinical manifestations, characteristic biochemical abnormalities, particularly metabolic acidosis, hyperparathyroidism, and reduced TmP/GFR, enable accurate etiological diagnosis and appropriate treatment. Based on these findings, we propose a hypothesis-generating clinico-biochemical diagnostic algorithm (Figure 2) derived from this single-centre cohort to facilitate the systematic evaluation of children with refractory rickets. The algorithm has not been externally validated and should therefore be interpreted as a preliminary framework requiring validation in larger multicentre studies. Although the findings are limited by the single-centre design, the relatively small sample size, failure to achieve the calculated sample size (32 enrolled vs. 34 estimated), incomplete genetic confirmation, and short follow-up period, the rarity of refractory rickets limited recruitment despite the inclusion of all consecutive eligible patients during the study period. The small shortfall in sample size may have slightly reduced the statistical power to detect differences between etiological groups, particularly for less common etiologies such as PRTA and VDDR, thereby increasing the possibility of type II error. Consequently, nonsignificant findings should be interpreted with caution. Nevertheless, this study provides important regional evidence on the etiological spectrum and treatment outcomes of refractory rickets. Larger multicentre studies with adequate sample sizes and longer follow-up are required to validate the proposed diagnostic algorithm and further optimize the management of refractory rickets.

## Conclusions

DRTA was the predominant cause of refractory rickets in this cohort. Although clinical manifestations overlapped across etiologies, characteristic biochemical abnormalities facilitated accurate diagnosis. Early etiology-specific treatment resulted in universal radiological healing despite variable biochemical recovery, highlighting the importance of timely diagnosis and individualized management to optimize outcomes in children with refractory rickets.
